# Physician-assigned Diagnosis Versus Questionnaire-based Measures in Individuals with Nociceptive, Neuropathic, and Nociplastic Pain: A Mechanism-based Characterization of Chronic Pain Patients

**DOI:** 10.5041/RMMJ.10580

**Published:** 2026-07-31

**Authors:** Netanel Kamel, Tseela Hoffman, May Haddad, Amir Minerbi

**Affiliations:** 1 Ruth and Bruce Rappaport Faculty of Medicine, Technion–Israel Institute of Technology, Haifa, Israel; 2 The Rambam Institute for Pain Medicine, Rambam Health Care Campus, Haifa, Israel; 3 Clinical Research Institute at Rambam (CRiR), Haifa, Israel

**Keywords:** Chronic pain, fibromyalgia, neuropathic pain, nociceptive pain, nociplastic pain

## Abstract

**Background:**

The International Association for the Study of Pain (IASP) has defined three mechanisms for chronic pain: nociceptive, neuropathic, and nociplastic. Accurate differentiation between these mechanisms is essential for treatment selection but remains challenging due to symptom overlap and reliance on clinical judgment. Patient-reported questionnaires may support diagnosis, though their diagnostic accuracy relative to physician assessment has not been fully established.

**Objectives:**

This study aimed to investigate the prevalence and overlap of chronic pain mechanisms in a tertiary pain clinic population and to compare physician-assigned diagnoses with results from patient-reported questionnaires.

**Methods:**

This cross-sectional study recruited 89 adult patients with chronic pain from the Rambam Institute for Pain Medicine at Rambam Health Care Campus in Haifa, Israel. Each participant underwent a comprehensive physician evaluation based on the criteria of the International Classification of Diseases and Related Health Problems, 11th revision, and completed the Central Sensitization Inventory (CSI), PainDETECT, and 2016 American College of Rheumatology (ACR) Fibromyalgia Diagnostic Criteria surveys. Diagnostic agreement, sensitivity, specificity, positive predictive value (PPV), and negative predictive value (NPV) were calculated using physician diagnosis as the reference standard.

**Results:**

Physician diagnoses identified neuropathic pain in 51 patients (57.3%), nociceptive pain in 38 (42.7%), and nociplastic pain in 25 (28.1%), with substantial diagnostic overlap (32.6% with ≥2 mechanisms). The CSI demonstrated high sensitivity (92.0%) but low specificity (45.3%) for nociplastic pain. PainDETECT showed moderate accuracy for neuropathic pain (sensitivity 68.6%, specificity 52.6%) and higher specificity for nociceptive pain (86.3%). The 2016 ACR Fibromyalgia criteria provided a more balanced diagnostic profile (sensitivity 88.0%, specificity 68.8%).

**Conclusions:**

Questionnaire-based measures are useful for screening chronic pain mechanisms but demonstrate variable accuracy when compared to physician diagnoses, particularly in the context of overlapping pain phenotypes. Refinement of diagnostic thresholds, combined assessment strategies, and updated questionnaires may improve differentiation of pain subtypes and enhance clinical decision-making.

## INTRODUCTION

### Classification of Pain Mechanisms

Chronic pain affects approximately 20% of the global adult population, placing a significant burden on both individuals and healthcare systems.^1^ The International Association for the Study of Pain (IASP) has proposed a classification system that categorizes chronic pain into three primary types according to its underlying mechanism: nociceptive, neuropathic, and nociplastic pain.^2^

Nociceptive pain results from activation of sensory neurons in response to potentially harmful stimuli such as injury, inflammation, or infection. It is typically described as sharp, aching, or throbbing and plays a protective role by signaling actual or potential tissue damage to initiate healing responses.^3^

Neuropathic pain, in contrast, results from damage or dysfunction within the nervous system and is often described as burning, shooting, or electric shock-like in nature. Common causes include nerve injury, diabetes, multiple sclerosis, infections, toxins, or genetic disorders.^3^

Nociplastic pain is described as diffuse musculoskeletal pain, persistent fatigue, and non-restorative sleep.^4^ While its exact pathophysiology remains unclear, diagnosis is generally made through symptom assessment and the exclusion of other medical conditions, with fibromyalgia being a common associated condition.^5^

### Available Diagnostic Tools

Clinical diagnosis of chronic pain is typically based on the patient’s history and physical examination, with ancillary tests being somewhat useful in certain disorders. Neuropathic pain is diagnosed according to the International Classification of Diseases and Related Health Problems, 11th revision (ICD-11) of the World Health Organization (WHO) criteria for neuropathic pain.^4^ It classifies pain based on three key elements: pain located in a neuroanatomically plausible distribution; history of a lesion or disease affecting the somatosensory nervous system; and clinical signs or diagnostic test results confirming somatosensory system involvement (e.g. sensory loss, allodynia, or imaging/nerve studies).^4^ The ICD-11 further subcategorizes chronic neuropathic pain into seven distinct types, including both peripheral (e.g. diabetic neuropathy, trigeminal neuralgia) and central neuropathic pain conditions (e.g. post stroke pain, spinal cord injury).^4^

Nociplastic pain is diagnosed using the 2021 IASP criteria, with diagnosis based on first confirming chronic pain, then excluding nociceptive and neuropathic causes, and, finally, by assessing for clinical features consistent with altered nociception, such as diffuse pain, hyperalgesia, and allodynia. Additionally, surveys like the 2016 American College of Rheumatology Preliminary Diagnostic Criteria for Fibromyalgia and Measurement of Symptom Severity (Fibromyalgia ACR)^6^ and the Central Sensitization Inventory (CSI)^5^ are both tools used to screen for or diagnose nociplastic pain syndromes. The Fibromyalgia ACR focuses on two scales: the Widespread Pain Index (WPI) and the Symptom Severity Scale (SSS), allowing for a comprehensive assessment of fibromyalgia symptoms.^6^

### The Utility of Questionnaires in the Diagnosis of Chronic Pain

Despite updated diagnostic frameworks from the IASP, National Institute for Healthcare and Excellence,^7^ and ICD-11 for identifying nociceptive, neuropathic, and nociplastic pain, these current practices still rely heavily on clinical history and physical examination. This dependence introduces potential variability in diagnostic accuracy across clinicians. Standardized patient-reported tools, such as the PainDETECT and CSI, may offer value as adjunctive measures to support clinical judgment. However, while these instruments are useful for capturing patient symptoms, their ability to reliably differentiate between pain mechanisms and serve as objective diagnostic tools remains an area of ongoing evaluation.

A 2011 study found that patients with diabetic neuropathy and fibromyalgia have similar sensory complaints, like burning, prickling, and allodoynia.^8^ Similarly, a 2013 study concluded that individuals with fibromyalgia should be assessed primarily through clinical evaluation rather than tools like PainDETECT,^9^ noting potential mechanistic overlaps between the conditions.^10^ These shared symptom profiles in patient-reported outcomes raise critical questions about the underlying pathophysiological commonalities across pain types and the use of surveys in clinical practice. This highlights the need for more refined sensory profiling and the development of precise terminology to better characterize, report, and differentiate between these complex pain types.

### Research Question

The primary aim of this study was to evaluate the agreement between physician-assigned diagnoses and the questionnaire-based patient-reported clinical measures.

## METHODS

### Study Design and Oversight

This observational, cross-sectional study took place at the Rambam Institute for Pain Medicine (Haifa, Israel). The study was approved by the Institutional Review Board of Rambam Health Care Campus (approval no. RMB-0010-23).

### Patient Recruitment and Clinical Evaluation

Participants were recruited from the Rambam Institute for Pain Medicine at Rambam Health Care Campus and equally divided into three diagnostic groups: 30 individuals with confirmed neuropathic pain, 30 with confirmed nociceptive pain, and 29 with a confirmed diagnosis of fibromyalgia. Inclusion criteria required participants to be adults aged 18 years or older who had been diagnosed by a pain specialist with fibromyalgia, nociceptive pain, or neuropathic pain according to the ICD-11 chronic pain classification. Participants needed to be able to read and sign an informed consent form. Individuals were excluded if their pain was of oncologic origin.

All participants were given a detailed explanation of the study and signed an informed consent form. They were evaluated by a specialized pain physician who completed a thorough assessment, including history and physical exam. The medical evaluation included documentation of each diagnosis using a mechanism-based approach in accordance with the ICD-11.

In addition, all participants completed three validated questionnaires: the Fibromyalgia ACR,^7^ PainDETECT,^9^ and the CSI.^11^,^12^ Predefined threshold scores indicating a high likelihood of diagnosis were used for comparison with physician-assigned diagnoses. A PainDETECT score ≥19 was considered indicative of a likely neuropathic pain diagnosis, while a score of ≤13 suggested a likely nociceptive pain profile. The Fibromyalgia ACR defined a likely diagnosis of fibromyalgia as meeting one of two score combinations: a WPI ≥7 and SSS ≥5, or WPI ≥4 and SSS ≥9. In addition, a CSI score ≥40 was used to identify individuals with nociplastic pain. Descriptive and anthropometric data, including age, sex, pain duration, stature, weight, and BMI, were also collected to characterize the study cohort.

### Data Analysis

Survey responses were recorded using REDCap software and scored in Microsoft Excel. Participants meeting the diagnostic threshold criteria were compared to those clinically diagnosed by a physician, using confusion matrices with the physician’s diagnosis serving as the reference standard. Sensitivity, specificity, positive predictive value (PPV), and negative predictive value (NPV) were calculated to compare diagnostic results. Statistical analyses were conducted using Microsoft Excel. *P* values <0.05 were considered statistically significant.

Demographic and clinical variables were evaluated for normality of distribution using the Shapiro–Wilk test. Normally distributed variables were compared using one-way ANOVA test, while non-normally distributed variables were compared using the Kruskal–Wallis test. Adjustment for multiple comparisons was performed using the Benjamini–Hochberg false discovery rate correction. Analyses were done on IBM^®^ SPSS® Statistics version 28.

## RESULTS

### Demographic Characteristics of Study Participants

A total of 89 patients with chronic pain disorders were included in the study. Table 1 provides demographic data and background information related to all study participants.

**Table 1:** Demographic Measures of Study Participants (*n*=89).

Variable	Value
Age (y), avg. (SD)	55.4 (13.3)

Weight (kg), avg. (SD)	78.5 (17.4)

Height (cm), avg. (SD)	170.1 (9.5)

BMI, avg. (SD)	27.1 (5.3)

Sex, *n* (%)	
Male	48 (53.9)
Female	41 (46.1)

Ethnicity, *n* (%)	
Israeli-Jewish	72 (80.9)
Israeli-Muslim	6 (6.7)
Israeli-Christian	6 (6.7)
Israeli-Druze	5 (5.6)

Diet type, *n* (%)	
Regular	82 (92.1)
Vegetarian	5 (5.6)
Vegan	1 (1.1)
Pescatarian	1 (1.1)

Marital status, *n* (%)	
Married or common-law	62 (69.7)
Separated or divorced	13 (14.6)
Single	11 (12.4)
Widowed	3 (3.4)

Smoking status, *n* (%)	
Never smoked	44 (49.4)
Currently smoking	23 (25.8)
Smoked in the past	22 (24.7)

Educational status, *n* (%)	
High school	34 (38.2)
Undergraduate degree or higher	53 (59.6)
Other	2 (2.2)

avg, average; BMI, body mass index; SD, standard deviation.

### Physician-assigned Diagnoses

**Figure 1:**
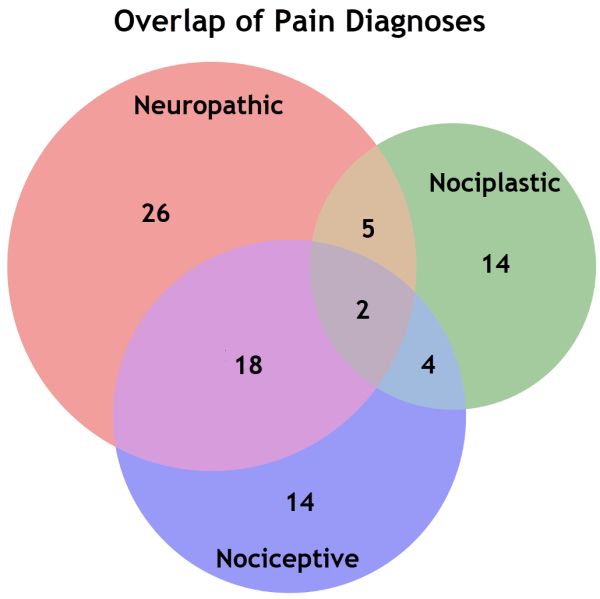
Physician-assigned Pain Mechanism Diagnoses and Their Overlap. Three-way Venn diagram showing patient counts for nociceptive, nociplastic, and neuropathic pain, including patients assigned multiple pain mechanism diagnoses.

There were overlapping physician-assigned diagnoses (Table 2, Figure 1). The most frequently assigned physician pain diagnoses were fibromyalgia syndrome, chronic painful radiculopathy, and chronic primary low back pain (Table 3). Other less commonly diagnosed pain conditions were chronic neuropathic pain after peripheral nerve injury, chronic primary musculoskeletal pain, and chronic secondary musculoskeletal pain associated with osteoarthritis.

**Table 2:** Distribution of Physician-assigned Pain Mechanisms Among Study Participants (*n*=89).

Pain Mechanism	Frequency	%
Neuropathic	51	57.3
Nociplastic	25	28.1
Nociceptive	38	42.7
Other	12	13.5

Percentages do not sum to 100% due to overlapping physician-assigned mechanisms.

**Table 3:** Frequency of Physician-assigned Pain Diagnoses.

Pain Condition	Number of Participants (*n*=89)
Fibromyalgia syndrome	18
Chronic painful radiculopathy	18
Chronic primary low back pain	17
Chronic neuropathic pain after peripheral nerve injury	15
Chronic primary musculoskeletal pain	14
Chronic secondary musculoskeletal pain associated with osteoarthritis	13
Complex regional pain syndrome	5
Spinal stenosis	5
Chronic painful polyneuropathy	4
Rheumatoid arthritis (inflammatory arthritis)	4
Chronic primary cervical pain	4

### Questionnaire Scores and Diagnoses

The mean CSI, PainDETECT, and ACR 2016 WPI and SSS scores for participants with physician-diagnosed nociceptive, neuropathic, and nociplastic pain are provided in Table 4.

**Table 4:** Patient-reported Questionnaire Mean (Standard Deviation) Scores by Physician-assigned Pain Mechanism.

Questionnaire	Nociceptive	Neuropathic	Nociplastic	*P* Value
CSI	41.9 (18.0)	45.5 (19.0)	60.8 (13.6)	<0.001*
PainDETECT	15.4 (9.4)	20.6 (9.5)	23.4 (5.7)	<0.001†
ACR 2016 (WPI)	6.1 (3.9)	6.4 (4.5)	10.8 (4.8)	0.002†
ACR 2016 (SSS)	5.7 (3.3)	6.6 (3.1)	8.9 (2.0)	0.002†

* One-way ANOVA.

† Kruskal-Wallis test.

For the PainDETECT survey, based on the diagnostic threshold of ≥19, participants with physician-diagnosed neuropathic and nociplastic pain met the questionnaire diagnostic threshold on average, while those with nociceptive pain did not. Furthermore, patients with physician-diagnosed nociceptive pain scored higher (on average) than patients with physician-diagnosed neuropathic pain on the PainDETECT (Table 4).

Patients with physician-diagnosed nociplastic pain had the highest mean scores across all survey tools (Table 4). On average, this group surpassed diagnostic thresholds for central sensitization (CSI≥ 40), neuropathic pain (PainDETECT≥19), and fibromyalgia/nociplastic pain (WPI≥7 and SSS≥5, or WPI≥4 and SSS≥9).

One-way ANOVA revealed a statistically significant difference in CSI scores among the three groups (*P*<0.001), with the physician-diagnosed nociplastic group reporting substantially higher values. On average, patients in all three groups exceeded the diagnostic threshold of CSI≥40, resulting in a high sensitivity of 92.0% but a low specificity of 45.3% for identifying nociplastic pain based on this cutoff (Table 5).

**Table 5:** Confusion Matrix of Physician and Survey-based Diagnoses of Nociplastic Pain (CSI score ≥40).

	Physician Positive Diagnosis	Physician Negative Diagnosis	

Questionnaire Positive Diagnosis	23	35	PPV=39.7%

Questionnaire Negative Diagnosis	2	29	NPV=93.5%
	
	Sensitivity=92.0%	Specificity=45.3%	


Physician-assigned diagnoses were used as the reference standard.

Regarding the Fibromyalgia ACR criteria, participants with physician-diagnosed nociplastic pain had the highest average scores on both the WPI and SSS (Table 4). The WPI and SSS values exceeded both diagnostic thresholds required for a survey-based fibromyalgia diagnosis (i.e. WPI≥7 and SSS≥5, or WPI≥4 and SSS≥9). On average, only the physician-diagnosed nociplastic pain group met full questionnaire-based criteria for fibromyalgia, demonstrating a good tool for both sensitivity and specificity (Table 6).

**Table 6:** Confusion Matrix of Physician and Survey-based Diagnoses of Nociplastic Pain (Fibromyalgia ACR score: WPI≥7 and SSS≥5, or WPI≥4 and SSS≥9).

	Physician Positive Diagnosis	Physician Negative Diagnosis	

Questionnaire Positive Diagnosis	22	20	PPV=52.4%

Questionnaire Negative Diagnosis	3	44	NPV=93.6%

	Sensitivity=88.0%	Specificity=68.8%	


Physician-assigned diagnoses were used as the reference standard.

### Agreement Between Physician Diagnosis and Questionnaires

For neuropathic pain, survey diagnoses based on a PainDETECT score of ≥19 yielded a sensitivity of 68.6% and a specificity of 52.6% when compared to physician diagnoses. The PPV and NPV values indicated moderate agreement between the survey tool and clinical diagnosis (Table 7).

**Table 7:** Confusion Matrix of Physician and Survey-based Diagnoses of Neuropathic Pain (PainDETECT Score ≥19).

	Physician Positive Diagnosis	Physician Negative Diagnosis	

Questionnaire Positive Diagnosis	35	18	PPV=66.0%

Questionnaire Negative Diagnosis	16	20	NPV=55.6%

	Sensitivity=68.6%	Specificity=52.6%	


Physician-assigned diagnoses were used as the reference standard.

For nociceptive pain, defined as a PainDETECT score ≤13, the results suggest that the survey tool had a relatively high specificity, indicating a better ability to rule out participants without physician-diagnosed nociceptive pain (Table 8).

**Table 8:** Confusion Matrix of Physician and Survey-based Diagnoses of Nociceptive Pain (PainDETECT score ≤13).

	Physician Positive Diagnosis	Physician Negative Diagnosis	

Questionnaire Positive Diagnosis	20	7	PPV=74.1%

Questionnaire Negative Diagnosis	18	44	NPV=71.0%

	Sensitivity=52.6%	Specificity=86.3%	


Physician-assigned diagnoses were used as the reference standard.

For nociplastic pain, when defined by a Central Sensitization Inventory (CSI) score ≥40, the survey tool results indicated that it was more effective in ruling out fibromyalgia, than in confirming it (Table 5).

Data from an alternative assessment of fibromyalgia pain using the Fibromyalgia ACR (which defines fibromyalgia pain as WPI ≥7 and SSS ≥5, or WPI ≥4 and SSS ≥9) indicated that the fibromyalgia-based criteria may offer a more balanced sensitivity and specificity diagnostic profile compared to the CSI (Table 6).

## DISCUSSION

This study compared the physician-assigned diagnoses of 89 chronic pain patients to their respective questionnaire-based measures.

When considering the physician-assigned diagnoses, 60.7% of patients had single-mechanism diagnoses, while 32.6% were diagnosed with two or more pain mechanisms. Neuropathic and nociceptive pain were the mechanisms that most commonly overlapped, occurring in 18 participants (Figure 1). Additional overlaps included 5 participants with both neuropathic and nociplastic pain, 4 with nociplastic and nociceptive pain, and 2 participants diagnosed with all three pain types: neuropathic, nociplastic, and nociceptive (Figure 1). The frequent co-occurrence of neuropathic and nociceptive pain may reflect true mechanistic overlap, or mere co-occurrence of unrelated conditions.

A total of 58 participants across all pain groups (65.2%) scored above the diagnostic threshold (>40) for CSI. The high sensitivity (92.0%) yet very low specificity (45.3%) of the CSI shows that while this questionnaire is effective at identifying individuals with nociplastic pain, it performs poorly at excluding those with other pain types (Table 5). Although all groups exceeded the CSI diagnostic threshold (≥40) on average, a one-way ANOVA revealed a statistically significant difference in mean scores (*P*<0.001), with the nociplastic group demonstrating a mean of 60.8±13.6. This suggests that while the current threshold offers high sensitivity, it may lack specificity for differentiating nociplastic pain, and higher scores may reflect more distinct nociplastic pain profiles. One possible explanation is that individuals with chronic pain may inherently exhibit elevated CSI scores, which could lead to false positives, particularly in patients with overlapping or comorbid conditions. These findings highlight the potential need to revise the diagnostic threshold to a higher value or to use the CSI in combination with other assessment tools to improve its balance between sensitivity and specificity.

PainDETECT appears to be effective in distinguishing nociceptive pain from neuropathic and nociplastic pain, but it performs poorly in differentiating between the latter two. A cutoff score of ≤13 results in a specificity of 86.3% and is effective at excluding neuropathic and nociplastic pain (Table 8). This finding aligns with the average PainDETECT score of 15.4±9.4 for nociceptive pain, which is lower than the mean 20.6±9.5 for neuropathic pain and 23.4±5.7 for nociplastic pain (*P*<0.001) (Table 4). Nonetheless, it still does not appear to be an effective screening tool for nociceptive pain as the sensitivity is 52.6% (Table 8). Taken together, these observations suggest that symptom burden frequently extends beyond a single pain mechanism and that multiple mechanisms may coexist within the same patient. This overlap likely reflects both the limited discriminatory specificity of individual questionnaires and the inherent complexity of chronic pain, in which nociceptive, neuropathic, and nociplastic processes often coexist rather than occur in isolation.

## CONCLUSION

The results of this study show that while questionnaires remain an imperfect diagnostic tool, they still can be a good screening tool for patients with nociplastic, nociceptive, or neuropathic pain. Furthermore, the common co-occurrence of multiple different pain mechanisms in patient populations and the high reporting of pain among patients with chronic pain highlight the need for measures to avoid overlap and decreased specificity. One potential solution can be to create a survey tool that uses a vocabulary with not only additive, but also deductive scoring—reducing the likelihood of diagnosing a condition when a patient’s complaints align more with alternative pain diagnoses. Additionally, while surveys have historically only been used to complement a physician’s physical exam, the exponential increase in use of artificial intelligence may soon transform this approach. The development of a more accurate diagnostic tool could enable artificial intelligence to make more reliable diagnoses, particularly when a physician may not be available.

## Abbreviations

ACR: American College of Rheumatology
CSI: Central Sensitization Inventory
Fibromyalgia ACR: 2016 American College of Rheumatology Preliminary Diagnostic Criteria for Fibromyalgia and Measurement of Symptom Severity
IASP: International Association for the Study of Pain
ICD-11: International Classification of Diseases and Related Health Problems, 11th revision
NPV: negative predictive value
PPV: positive predictive value
